# Ekbom Syndrome - A Case Report

**DOI:** 10.1192/j.eurpsy.2023.2257

**Published:** 2023-07-19

**Authors:** K. Keenan, C. Clarke

**Affiliations:** Department of Psychiatry of Old Age, CHO9 HSE Dublin North City Central Mental Health Services, Dublin, Ireland

## Abstract

**Introduction:**

Ekbom Syndrome is a rare condition presenting as a uni-thematic delusional belief of parasitosis. Affected individuals are often socially isolated presenile females. The syndrome is characterised by kinaesthetic hallucinations of insect infestation with persistent pruritis. First described by Karl Ekbom in 1938, presentations are rare, often presenting initially to primary care and dermatological services. We report a case of an older adult female referred to Community Mental Health Team following multiple presentations to the primary care physician with subsequent diagnosis of Ekbom Syndrome.

**Objectives:**

To illustrate a rare case report of Ekbom Syndrome, managed successfully in the community in Ireland.

**Methods:**

A retrospective case study. Data was reviewed from available psychiatric and medical records including laboratory testing.

**Results:**

A 69 year old Catholic Nun was referred to the CMHT for psychiatric assessment. The patient presented to the CMHT appointment as distressed. She reported a 15 year history with significant deterioration 4 months prior, of progressive symptoms of a ‘crawling and biting sensation’ all over her body, alongside intermittent anxiety related to the infestation. The patient acknowledged that she had visited her primary care physician on multiple occasions seeking resolution. On one occasion the patient brought a sample of the alleged parasites inside a small container, ‘matchbox sign’.

A professional pest control agency had recently been employed to decontaminate her bedroom in the parish house of which she is resident in. She described a rigid routined, daily washing of clothes. Medical history was significant for pituitary gland adenoma 30years prior, with pituitary excision twice secondary to visual disturbance and reoccurrence. She was on lifelong thyroid replacement with acceptable postoperative functioning.

The patient was commenced on Aripiprazole oral medication and received psychoeducation via Specialist Mental Health Nursing Outreach. Pimozide was deemed unsuitable due to presence of numerous cardiac risk factors. Physical and delusional psychopathology resolved in a trajectory fashion with aripiprazole titration. It was hypothesised that the patient had premorbid anakastic personality traits, exacerbated by the COVID19 pandemic prior to presentation.

**Conclusions:**

Patients with delusional parasitosis can have complex medical and social histories and may present to psychiatry as a last resort. These clinical presentations can occur after periods of dermatological input and following extensive efforts to decontaminate their physical surroundings. Psychotic symptoms of Ekbom Syndrome may be effectively controlled with psychotropic therapy and patients benefit from psychoeducation about their rare disorder.

**Disclosure of Interest:**

None Declared

